# Simultaneous removal of methylene blue and Cr(VI) in a dual-chamber photocatalytic microbial fuel cell with WO_3_/MoS_2_/FTO photocathode

**DOI:** 10.1016/j.heliyon.2024.e29204

**Published:** 2024-04-10

**Authors:** Jiye Xin, Shishi Kong, Xiaoliang Zhang, Yujuan Yang, Xuan Wang

**Affiliations:** a School of Ecology and Environment, Inner Mongolia University, 24 Zhaojun Road, Hohhot, Inner Mongolia, 010070, China; b Key Laboratory of Environmental Pollution Control and Waste Recycling, Inner Mongolia Autonomous Region, 24 Zhaojun Road, Hohhot, Inner Mongolia, 010070, China

**Keywords:** Photocatalytic microbial fuel cell, WO_3_/MoS_2_/FTO photocathode, Methylene blue, Cr(VI), Z-scheme heterojunction

## Abstract

Carbon felt was used as the anode and WO_3_/MoS_2_/FTO (fluorine-doped tin oxide) was used as the photocathode in a photocatalytic microbial fuel cell (PMFC). The photoelectric performance of the WO_3_/MoS_2_/FTO photocathode and the removal efficiency of methylene blue (MB) and Cr(VI) mixed pollutants were systematically investigated in the cathode chamber. The results showed that after 12 h of light irradiation in the PMFC with WO_3_/MoS_2_/FTO as the photocathode, the removal rates of MB and Cr(VI) were 84.56 and 68.11 %, respectively, which were much higher than those using WO_3_/FTO as a photocathode (55.57 % and 45.26 %, respectively). The corresponding maximum output power was 33.14 mW/m^2^, which was 1.85 times that of the WO_3_/FTO photocathode PMFC. These results can be attributed to the fact that WO_3_ is an n-type semiconductor and MoS_2_ is a p-type semiconductor. Analysis of trapping experiments showed that the composite of WO_3_ and MoS_2_ formed a Z-scheme heterojunction, which improved the separation efficiency of the photoelectric carriers and enhanced the pollutant removal efficiency of the photocathode. PMFCs are a new and environment-friendly technology for removing pollutants thereby providing an experimental basis for future engineering applications.

## Highlights

• A p-n/z-type heterojunction was formed between methylene blue and Cr(VI).
• A coupled system of biological anode and photocathode was constructed.
• Methylene blue and Cr(VI) have synergistic removal effects.

## 1 Introduction

With rapid industrial development around the world, pollutants discharged into the environment are becoming increasingly toxic and chemically complex [1]. Many industrial wastewaters contain both heavy metal ions and organic dyes, which can cause serious harm to the environment and humans [2]. Methylene blue (MB) is a commonly used cationic dye that is chemically stable and can affect the growth of aquatic plants and microorganisms, thus causing serious ecological problems [3], and exposure to MB may also cause human health effects such as cyanosis, shock, jaundice, vomiting, necrosis, and quadriplegia [4,5]. Cr in effluents mainly exists in the forms of Cr(VI) and Cr(III), and Cr(VI) is highly toxic to most organisms and has carcinogenic, teratogenic, and mutagenic effects [6,7]. MB and Cr(VI) are simultaneously present in wastewater from the pigment, printing, dyeing, leather, metallurgical, cosmetic, and electroplating industries [8]. Therefore, effective methods for removing organic pollutants and heavy metals have attracted the attention of many scientists and engineers in the field of environmental chemistry.

Recently, various methods have been developed to treat mixed wastewater, including chemical precipitation, ion exchange, membrane separation, adsorption, coagulation, and advanced oxidation. However, conventional treatment technologies introduce other chemicals, resulting in additional energy consumption and secondary pollution [9,10]. Microbial fuel cell (MFC) technology has received increasing attention owing to its renewable, clean, and environment-friendly characteristics [11]. However, the main drawbacks of MFC include low power density, high cathodic overpotential, and poor wastewater treatment efficiency [12]. In addition, microbial activity is highly influenced by environmental factors, such as temperature, pH, and light. Therefore, synergistic systems have been proposed to improve wastewater treatment efficiency [13]. Photocatalysis (PC) is a method to degrade organic pollutants by generating strong oxidants [14,15]. However, high carrier complex efficiency limits the development of photocatalysis [16]. Photocatalytic microbial fuel cell (PMFC) systems effectively combine MFCs with photocatalysis. In these coupled systems, the MFC provides a low voltage to reduce electron–hole complexation in the photocathode, thereby increasing pollutant removal efficiency [17]. Touach et al. used BaTiO_3_ functional chalcocite as a photocathode in a microbial fuel cell for wastewater treatment and energy production, which ultimately increased the maximum power density and open-circuit voltage of an MFC system [18]. Chen et al. coupled Ce-g-C_3_N_4_ photoelectrodes and a multistage microbial fuel cell for the treatment of highly concentrated saline amine-enriched industrial wastewater to remove 88 % of the chemical oxygen demand (COD) [19]. In a PMFC, the photogenerated holes on the photocathode can combine with electrons generated from the bioanode instead of photogenerated electrons [20]; thus, the complexation of photogenerated electron–hole pairs can be effectively inhibited, enhancing pollutant removal efficiency. In addition, because the photocatalyst is fixed to the cathode, PMFC can solve the problem of photocatalyst recovery [21]. Therefore, the preparation of suitable photocathode materials is crucial to improve the performance of PMFCs.

Tungsten trioxide (WO_3_) is an n-type semiconductor material that has been widely studied owing to its good chemical stability, nontoxicity, light corrosion resistance, and abundant resource reserves. The preparation process is simple, and its other advantages have been widely studied [22]. However, WO_3_ suffers from a high photogenerated electron–hole pair complexation rate, the ability to absorb a limited range of light, and a low conduction band position, thereby limiting its application in photocatalysis [23]. Heterojunction construction is an effective method of improving the photocatalytic performance of semiconductor materials. Molybdenum disulfide (MoS_2_) is an emerging graphene-like layered compound, with layers maintained by van der Waals forces between layers and a molecular structure with a large number of S atoms exposed on the surface, and has high surface activity [24,25]. In addition, MoS_2_ has excellent light absorption, photothermal properties, and reduction abilities [26,27]. The WO_3_ and MoS_2_ energy band structures match well, which is in line with the design mechanism of heterojunctions in a Z-scheme system [28]. Using the built-in electric field between WO_3_ and MoS_2_, photogenerated carriers can be effectively separated, thus enhancing the removal of pollutants.

In this study, a PMFC-coupled system was constructed using WO_3_/MoS_2_/FTO (fluorine-doped tin oxide) as the photocathode and the structural and optical properties of the photocathode materials in the PMFC system were investigated. The synergistic degradation and power production of MB and Cr(VI) by the PMFC system were explored, and the effects of catalyst loading, pH, and reaction kinetics are discussed. Finally, the reactive species involved in MB and Cr(VI) removal were analysed, and possible reaction mechanisms were explored. This study provides new directions for the future treatment of wastewater in the environmental field.

## 2 Materials and methods

### 2.1 Materials

FTO was purchased from Luoyang Guluo Glass Co, (Luoyang, China). Sodium tungstate (Na_2_WO_4_·2H_2_O), sodium molybdate (Na_2_MoO_4_·2H_2_O), and thiourea (CH_4_N_2_S) were purchased from Sinopharm Chemical Reagent Co. (Shanghai, China). Sodium chloride (NaCl), concentrated hydrochloric acid (HCl), and glucose (C_6_H_12_O_6_) were purchased from the Tianjin Damao Chemical Reagent Factory (Tianjin, China). The above chemicals were of analytical grade and were used without further purification. Deionised (DI) water was used throughout the study.

### 2.2 Synthesis of WO_3_/MoS_2_/FTO photocathode

Step 1: Synthesis of WO_3_. Approximately 3 g of Na_2_WO_4_·2H_2_O and 1.5 g of NaCl were dissolved in 60 mL of deionised water, and this mixture was sonicated for 60 min to obtain a homogeneous solution. The pH of the solution was adjusted to 2 with 4 M HCl. The resulting solution was transferred to a Teflon-lined stainless-steel autoclave (200 mL) and heated at 180 °C for 24 h. After cooling to room temperature, the prepared material was rinsed three times with anhydrous ethanol and deionised water and then dried in a blast drying oven at 60 °C, producing a pale white WO_3_ powder.

Step 2: Synthesis of MoS_2_. In 60 mL of deionised water, 2 g of Na_2_WO_4_·2H_2_O and 4 g of C_2_H_5_NS were dissolved. The mixture was then sonicated for 60 min to obtain a homogeneous solution, which was then transferred to a Teflon-lined stainless-steel autoclave (200 mL) and heated at 180 °C for 12 h. After cooling to room temperature, the prepared material was rinsed three times with anhydrous ethanol and deionised water and then dried in a blast drying oven at 60 °C, producing black MoS_2_ powder.

Step 3: Synthesis of MoS_2_/WO_3_. To 60 mL of 0.5 M glucose solution, 1 g of WO_3_ was added and the mixture was sonicated for 60 min. Then, 1 g Na_2_MoO_4_·2H_2_O and 2 g C_2_H_5_NS were dissolved in the mixture for in-situ production of MoS_2_ and sonicated for 60 min to obtain a homogeneous solution, which was transferred to a 200 mL Teflon-lined stainless-steel autoclave and heated at 180 °C for 12 h. After cooling to room temperature, the prepared materials were rinsed three times with anhydrous ethanol and deionised water and then dried in a blast drying oven at 60 °C [29,30].

Step 4: Pre-treatment of FTO. Several 2 cm × 2 cm FTO samples were pre-treated with ethyl acetate, acetone, anhydrous ethanol, and deionised water with ultrasonication for 1 h and then placed in a blower drying oven at 60 °C to be dried and used as a substrate.

Step 5: Preparation of electrodes. Twenty mg of powdered material were prepared by the hydrothermal method and 0.2 mL of anhydrous ethanol and 0.12 mL of 5 % Nafion solution were added. The mixture was then subjected to ultrasonic treatment for 10 min to obtain a uniform solution. The powder dispersion was repeatedly applied onto a conductive glass using a rubber-tipped burette and then dried in a 60 °C blast drying oven.

### 2.3 Construction and operation of PMFC

**Fig. 1:**
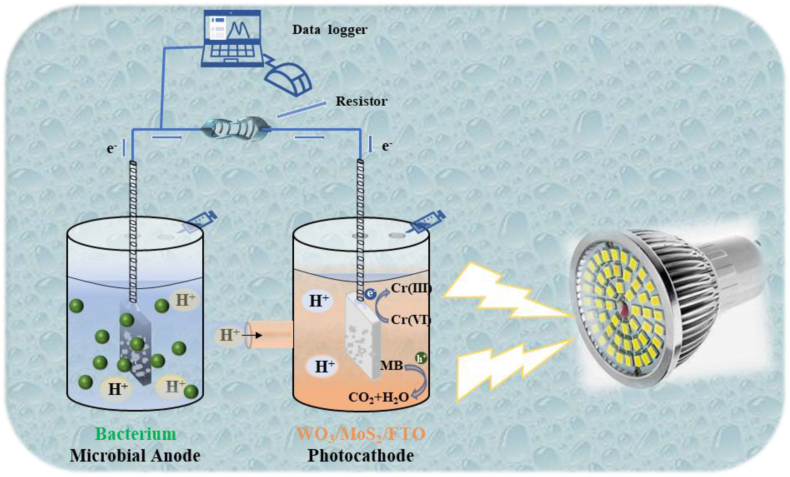
Schematic diagram of PMFC system.

Fig. 1 Shows a schematic diagram of the PMFC reactor used in this experiment. The PMFC reactor consists of two identical cylindrical chambers, each with a total volume of 200 mL, connected to a proton exchange membrane (PEM). Carbon felt (2 × 2 × 0.6 cm^3^) is used as the anode, and WO_3_/MoS_2_/FTO as the photocathode. An external resistor is connected between the two electrodes using a wire, and the distance between the PEM and each electrode is approximately 4.0 cm. A configured liquid medium is used as the carbon source for microorganisms in the anode chamber. The cathode chamber contains 100 mL of a mixed solution of MB and Cr(VI) at a concentration of 10 mg/L as the target contaminant.

### 2.4 Analytical methods

The morphology and phase of the WO_3_/MoS_2_ were characterised using field-emission scanning electron microscopy (FE-SEM, Thermo Fisher Quattor S, USA) and high-resolution transmission electron microscopy (HRTEM, Jem-2100F, USA). The phase structure and crystallinity of the samples were examined using an X-ray diffractometer (XRD, Shimadzu 6100, Japan) with a scanning range of 10°–90°, using a Cu Kα radiation source (40 Kv, 150 Ma). The surface chemical composition and electronic structure of WO_3_/MoS_2_ were examined using X-ray photoelectron spectroscopy (XPS, Nexsa, Thermo Fisher Scientific). A fluorescence spectrometer (Edinburgh FLS-1000) was used for photoluminescence (PL) spectroscopic studies at an excitation wavelength of 325 nm. Diffuse reflectance spectra (DRS) were obtained using a UV–visible spectrophotometer (Shimadzu 3600+, Japan) to analyse the optical absorption properties of WO_3_/MoS_2_ and calculate the bandgap energy.

Cyclic voltammetry (CV), I-t curves, and electrochemical impedance spectroscopy (EIS) were performed using an electrochemical workstation (Zennium Pro, Zahner). All experiments were conducted using a three-electrode system, with 0.1 M Na_2_SO_4_ solution as the electrolyte, WO_3_/MoS_2_/FTO as the working electrode, platinum as the counter electrode (CE), and Ag/AgCl as the reference electrode (RE). The CV tests were performed with a scan rate of 50 mV/s in the range −0.8 V–0.8 V. EIS tests were performed at a scan frequency of 1000 Hz to 5 MHz and an amplitude of 10 mV.

The concentration of MB was determined using a UV spectrophotometer (TU-1901; Beijing Puxi General Instrument Co., Ltd.) at an absorption wavelength of 664 nm. The Cr(VI) concentration was determined using diphenylcarbazide spectrophotometry at an absorption wavelength of 540 nm. Real-time voltage monitoring of the MFC was performed using a data collector (Picolog1216, Pico1216 Technologies Ltd, UK) and the battery voltage of the PMFC was recorded every 10 min. The voltages were recorded at different resistances by varying the amount of external resistance (10–90,000 Ω), and finally, the current and power densities were calculated according to Eqs. (1), (2), respectively:

(1) $$I=\frac{U}{R*A}$$

(2) $$P=\frac{U^{2}}{R*A}$$

where I is the current density (mA/m^2^), U is the voltage output (mV), R is the external resistance (Ω), A is the projected area of anode electrode (m^2^), and P (mW/m^2^) represents the power density.

The removal efficiencies (η) of MB and Cr(VI) were calculated using Eq. (3):

(3) $$\eta \left(\%\right)=\frac{C_{0}-C_{t}}{C_{0}}\times 100$$

where C_0_ and C_t_ are the concentrations (mg/L) initially and at time t, respectively.

## 3 Results and discussion

### 3.1 Characterisation of the photocathode

#### 3.1.1 Morphology analysis of photocatalysts

**Fig. 2:**
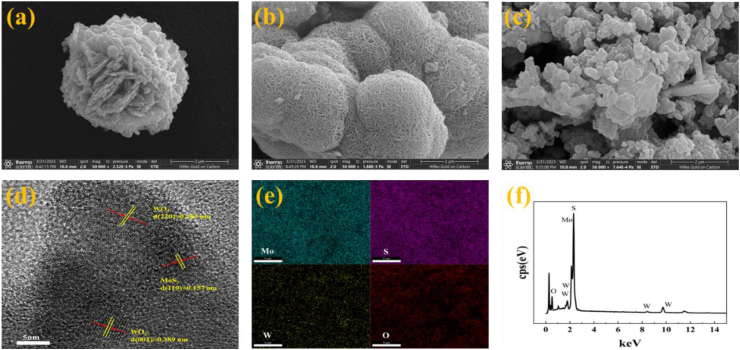
SEM images of (a)WO_3_, (b) MoS_2_, (c) WO_3_/MoS_2_, (d) HRTEM image of WO_3_/MoS_2_, and (e, f) EDS image of WO_3_/MoS_2_.

SEM was used to examine the morphology of the composites. Fig. 2 (a) shows an SEM image of WO_3_, and it can be seen that the nanoflower-like WO_3_ is composed of many WO_3_ nanosheets. Fig. 2 (b) shows an SEM image of MoS_2_, indicating spherical MoS_2_ particles composed of entangled nanofilaments. Fig. 2 (c) shows tight binding of the two components in the WO_3_/MoS_2_ semiconductor. HRTEM was used to further confirm the crystal structure and morphology of WO_3_/MoS_2_. In Fig. 2 (d), the lattice spacing calculated from the diffraction pattern is 0.157 nm, which corresponds to the (110) crystal plane of MoS_2_. The other two lattice spacings calculated from the diffraction pattern are 0.183 and 0.389 nm, which correspond to the (220) and (002) crystal planes of WO_3_, respectively. Successful preparation of the WO_3_/MoS_2_ composite is indicated. The energy-dispersive spectroscopy (EDS) profiles displayed in Fig. 2 (e) and (f) were used to investigate the elemental distribution, composition, and purity of the catalysts. Uniform distribution of W, O, Mo, and S in the WO_3_/MoS_2_ composites is observed via EDS spectral analysis, and no obvious clustering is observed. A uniform distribution of W, O, Mo, and S elements is also observed in the WO_3_/MoS_2_ composites without any obvious clustering.

#### 3.1.2 Structural properties of photocatalysts

**Fig. 3:**
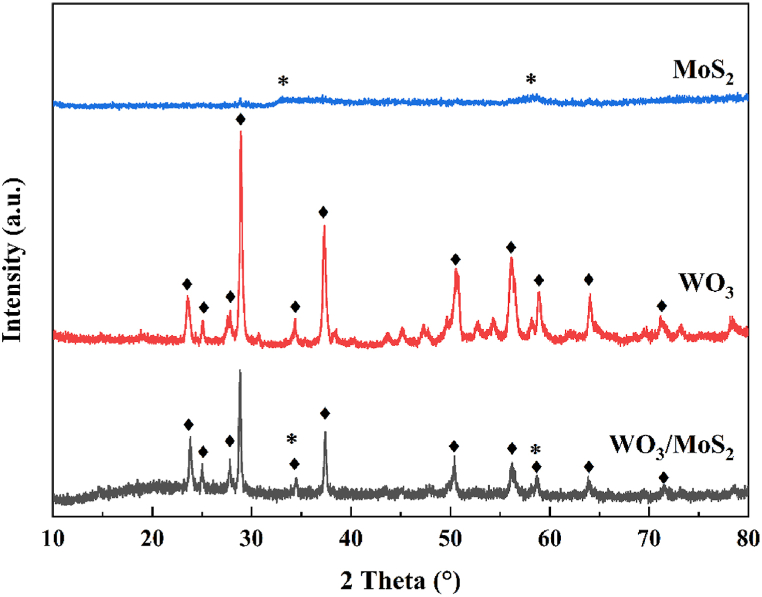
XRD spectra of MoS_2_, WO_3_, and WO_3_/MoS_2_.

The phase composition and crystallinity of the materials was analysed using XRD. As shown in Fig. 3, the crystallinity of WO_3_ is highlighted by the appearance of diffraction peaks for the (002), (110), (102), (200), (112), (202), (220), (222), (400), (402), and (224) crystal planes with corresponding 2θ values of 23.47°, 25.02°, 27.87°, 28.93°, 34.21°, 37.31°, 50.54°, 56.06°, 58.89°, 64.03°, and 71.24° (JCPDS 85–2460). The two broad peaks at 2θ = 32.92° and 58.07° for MoS_2_ are similar to those for the (100) and (110) crystal planes (JCPDS 77–1716), suggesting that the crystallinity of the synthesised MoS_2_ has poor crystallinity [31]. In the WO_3_/MoS_2_ composite semiconductor, most of the diffraction peaks are from WO_3._ The diffraction peaks of MoS_2_ are relatively insignificant, which may be due to the lower intensity of MoS_2_, and broadened owing to the amorphous nature of MoS_2_.

**Fig. 4:**
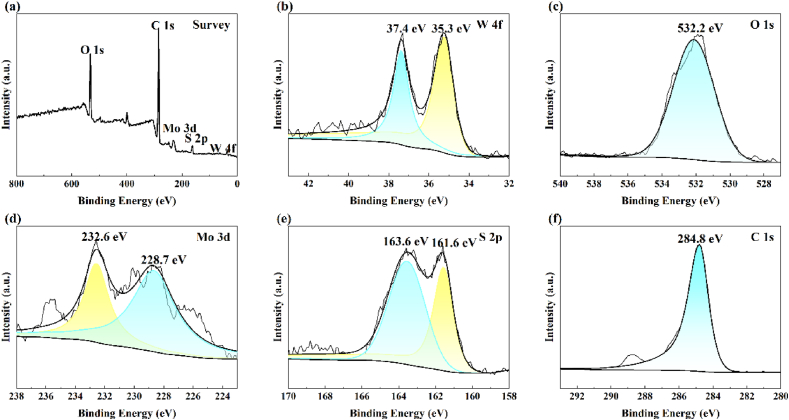
X-ray photoelectron spectroscopy of WO_3_/MoS_2_: (a) Full spectrum, (b) W 4f spectrum, (c) O 1s spectrum, (d) Mo 3d spectrum, (e) S 2p spectrum, and (f) C 1s spectrum.

The elemental composition and chemometrics of the composites were determined using XPS. Fig. 4 (a) shows that WO_3_/MoS_2_ has peaks at the W, O, Mo, and S positions, indicating the presence of these elements in the WO_3_/MoS_2_. Fig. 4 (b) shows the high-resolution W 4f spectrum of WO_3_/MoS_2_, with characteristic peaks located at 37.4 eV and 35.3 eV corresponding to the inner electrons of W 4f_5/2_ and W 4f_7/2_ [32]. The difference in the binding energies between the two peaks is approximately 2.1 eV, suggesting that W is in the valence state W^6+^. However, the characteristic peak of W is shifted to a slightly lower value than the reported value, which may be due to the presence of oxygen vacancies [33]. Fig. 4 (c) shows the high-resolution O 1s spectrum of WO_3_/MoS_2_, and the characteristic peak located at 532.2 eV is related to the W–O–W chemical bond. Fig. 4 (d) shows the high-resolution Mo 3d spectra of WO_3_/MoS_2_, where the characteristic peaks located at 232.6 eV and 228.7 eV correspond to the inner electrons of Mo 3d_3/2_ and Mo 3d_5/2_, respectively, while the peak appearing at 232.6 eV corresponds to Mo^6+^, and the peak at 228.7 eV is attributed to Mo^4+^ in MoS_2_ [34], which indicates that the surface of the WO_3_/MoS_2_ material is partially oxidised. Fig. 4 (e) shows the high-resolution S 2p spectrum of WO_3_/MoS_2_, and the characteristic peaks located at 163.6 eV and 161.6 eV correspond to the inner electrons of S 2p_1/2_ and S 2p_3/2_ [35], indicating the possible presence of S^2−^ in WO_3_/MoS_2_. The above results confirm the presence of WO_3_ and MoS_2_ in the prepared composite WO_3_/MoS_2_.

#### 3.1.3 Optical and photoelectric properties

**Fig. 5:**
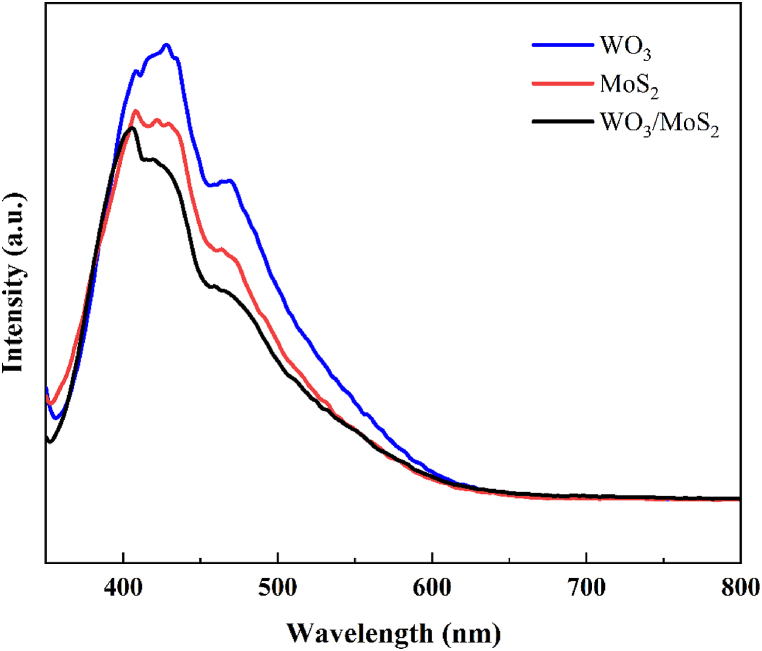
PL spectra of WO_3_, MoS_2_, and WO_3_/MoS_2_.

PL spectra can reveal the generation, separation, migration, and recombination of photogenerated charge carriers at the catalytic interface of materials [12]. The photocatalytic activity is inferred from the recombination ability of excitons, which is directly related to the PL intensity. As shown in Fig. 5, the fluorescence emission spectra of WO_3_, MoS_2_, and WO_3_/MoS_2_ were recorded at an excitation wavelength of 325 nm [36]. The fluorescence signal intensity of the WO_3_/MoS_2_ composite is significantly weaker than that of WO_3_ and MoS_2_ alone, which indicates that the recombination and compounding ability of photogenerated holes and electrons in the WO_3_/MoS_2_ composite is weakened; this can be attributed to the formation of heterojunctions in the binary composite material, which enhances the separation of charges.

**Fig. 6:**
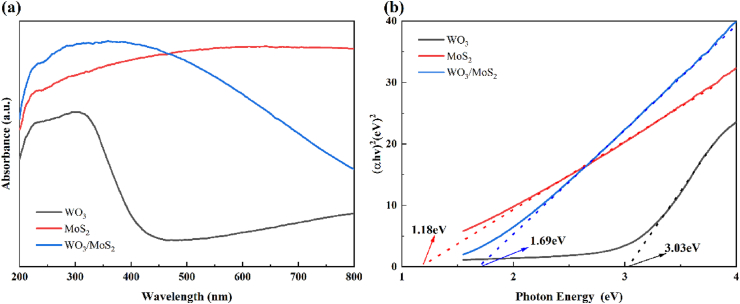
WO_3_, MoS_2_, and WO_3_/MoS_2_: (a) UV–Vis-DRS spectra and (b) Tauc diagram.

The optical properties and band gap energies of the synthesised catalysts were investigated using UV–vis DRS. Fig. 6 (a) shows that WO_3_ has poor light absorption in the visible range, whereas MoS_2_ exhibits a wide range of light absorption in the UV and visible regions. The spectral absorption boundary of WO_3_/MoS_2_ exhibits a systematic redshift due to the modification of MoS_2_. The bandgap energies (Eg) of the photocatalysts were calculated using Eq. (4):

(4) $${\left(\alpha hv\right)}^{2}=k\left(hv-E_{g}\right)$$

where α, h, ν, k, and Eg are the absorption coefficient, Planck's constant, incident photon frequency, proportionality constant, and band gap energy, respectively. Plotting with hν as the horizontal coordinate and (αhν)^2^ as the vertical coordinate, the Tauc plot of Fig. 6 (b) is obtained. Extrapolating a tangent line on the x-axis, the forbidden bandwidths of WO_3_, MoS_2_, and WO_3_/MoS_2_ are 3.03, 1.18, and 1.69 eV, respectively, which indicates that the addition of MoS_2_ lowers the bandgap of WO_3_ and contributes to the photocatalytic activity. To determine the energy band structure of WO_3_ and MoS_2,_ the E_VB_ and E_CB_ values of WO_3_ and MoS_2_ were calculated using Eqs. (5), (6) [37]:

(5) $$E_{VB}=X-E_{X}+\frac{1}{2}E_{g}$$

(6) $$E_{CB}=E_{VB}-E_{g}$$

where X is the geometric mean of the electronegativity of the semiconductor atoms (6.59 and 5.32 eV for WO_3_ and MoS_2_, respectively) and E_X_ is the energy of free electrons from the energy level of the standard hydrogen electrode (4.50 eV vs. NHE). The bandgap energy of WO_3_ is 3.03 eV, and the calculated valence and conduction bands are 3.605 and 0.575 eV, respectively; MoS_2_'s band gap energy is 1.18 eV and the calculated valence and conduction bands are 1.41 and 0.23 eV, respectively.

**Fig. 7:**
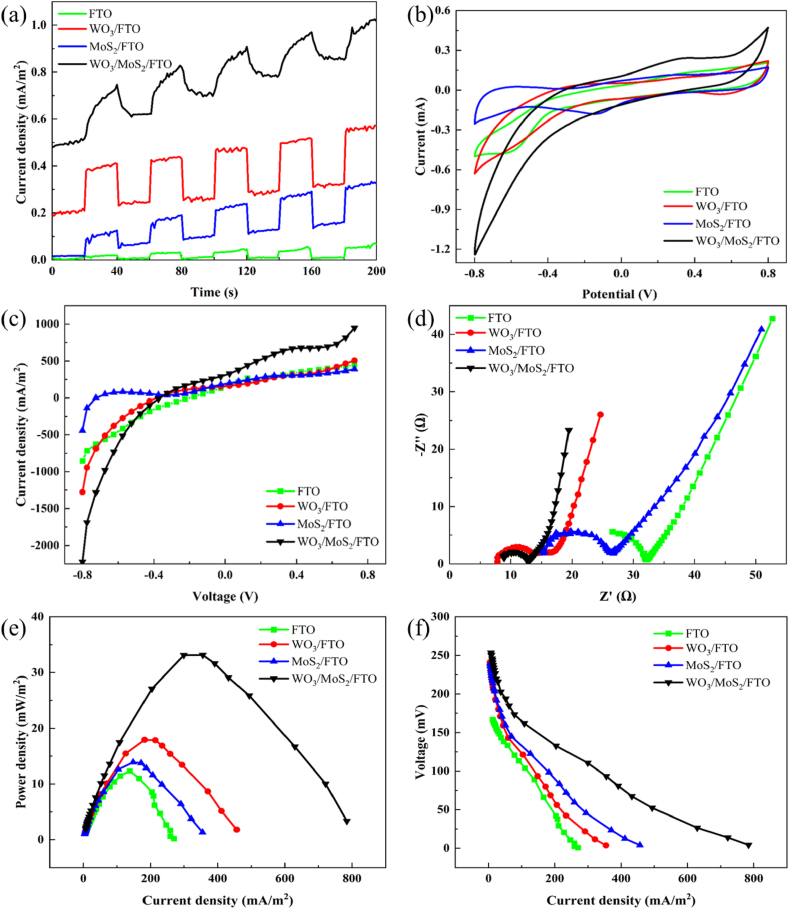
(a) Transient photocurrent response curves, (b) CV curves, (c) Linear voltammetry curves, (d) EIS Nyquist plot, (e) Power density curves, and (f) Polarisation curves.

Fig. 7 (a) shows the photocurrent density of the photocathode. The maximum photocurrent density of WO_3_/MoS_2_/FTO is 1.0251 mA/m^2^, which is 1.79, 3.08, and 18.60 times higher than those of WO_3_/FTO (0.5733 mA/m^2^), MoS_2_/FTO (0.3333 mA/m^2^), and FTO (0.0551 mA/m^2^), respectively. This indicates that combining MoS_2_ and WO_3_ improves the photoelectric performance by effectively separating photogenerated electrons and holes, thereby significantly improving the photocatalytic performance. In addition, it can be seen that the photocurrent generated by the WO_3_/MoS_2_/FTO photocathode has good reproducibility, indicating good stability. Fig. 7 (b) shows CV curves of four different photocathodes. The magnitude of the specific capacitance of different photocathodes can be determined by the integrated area under the closed CV curve, and that of WO_3_/MoS_2_/FTO is the largest, indicating that the WO_3_/MoS_2_/FTO photocathode has a larger electrochemically active surface area [38], which helps to enhance the charge transfer and electron transport efficiency in the cell system. Fig. 7 (c) shows the results of linear voltammetry tests on the different photocathodes. The WO_3_/MoS_2_/FTO photocathode exhibits the largest photocurrent response of −2225.1 mA/m^2^ at a potential of −0.8 V (vs. SHE), which can be attributed to rapid diffusion of the surface charge of the composite material [39]. EIS was used to evaluate the charge-transfer performance and internal resistance of different materials. As shown in Fig. 7 (d), WO_3_/MoS_2_/FTO has the smallest half-cycle arc radius in the Nyquist plot compared to those of the other samples, indicating that the composite material has the lowest interfacial charge-transfer internal resistance at the electrode/electrolyte interface, and the electron–hole separation efficiency of the photocathode WO_3_/MoS_2_/FTO is the highest [40].

Fig. 7 (e) and (f) show the power density and polarisation curves of the PMFC with different materials as photocathodes. The maximum power density of WO_3_/MoS_2_/FTO as photocathode is 33.14 mW/m^2^, which is 2.68, 1.85 and 2.39 times higher than that of FTO (12.33 mW/m^2^), WO_3_/FTO (17.94 mW/m^2^), and MoS_2_/FTO (13.89 mW/m^2^), respectively. The internal resistance of the PMFC system when the four materials were used as photocathodes was obtained by linear fitting of the polarisation curves. The internal resistances of FTO, WO_3_/FTO, MoS_2_/FTO, and WO_3_/MoS_2_/FTO were 1604.38, 1272.55, 1011.70, and 646.58 Ω, respectively. This indicates that the composite WO_3_ and MoS_2_ photocathode reduces resistance to electron transport in the cell system, which enhances the mass transfer rate and improves the catalytic efficiency of the materials [41].

### 3.2 Simultaneous removal of Cr(VI) and MB in PMFC

A mixed solution (100 mL) of 10 ppm each of MB and Cr(VI) was placed in the cathode chamber of the PMFC and reacted under 200 W LED light for 12 h. The influence of various factors on the removal of MB and Cr(VI) was investigated.

The rate constant was determined by Eq. (7):

(7) $$\ln\left(C_{0}/C\right)=kt$$

where C_0_ and C are the initial and final concentrations at time (t = 0) and time t respectively, and k is the rate constant.

#### 3.2.1 Effect of different photoelectrodes

**Fig. 8:**
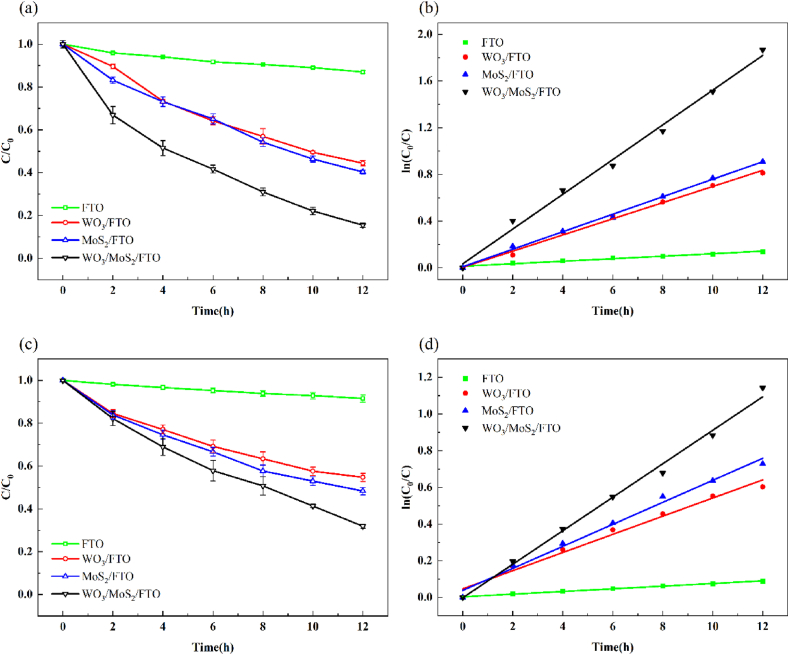
Effect of different photoelectrodes. (a) Degradation curve, (b) first order plots for the photodegradation of MB, (c) degradation curve, and (d) first order plots for the photoreduction of Cr(VI).

Fig. 8 shows the removal of MB and Cr(VI) from mixed wastewater using different photocathodes under the same reaction conditions. Table 1 lists the efficiency of MB and Cr(VI) removal and their corresponding reaction rate constants. Compared to the FTO, WO_3_/FTO, and MoS_2_/FTO photocathodes, the WO_3_/MoS_2_/FTO photocathode showed the best removal of mixed pollutants MB and Cr(VI) with removal efficiencies of 84.56 and 68.11 % and rate constants of 0.1487 and 0.0913 h^−1^, respectively. The WO_3_/MoS_2_/FTO photocathode exhibits a stronger effect on the removal of pollutants because of the formation of heterojunctions between WO_3_ and MoS_2_, which promotes the separation of electron–hole pairs and improves photocatalytic activity. Therefore, the WO_3_/MoS_2_/FTO photocathode was selected for subsequent experiments.

**Table 1:** Removal and reaction rate constants of mixed pollutants of MB and Cr(VI) under different photocathodes.

Photocathode	MB		Cr(VI)	
	*η* (%)	k (h^−1^)	*η* (%)	k (h^−1^)
FTO	12.98	0.0108	8.48	0.0072
WO_3_/FTO	55.57	0.0692	45.26	0.0495
MoS_2_/FTO	59.71	0.0749	51.70	0.0599
WO_3_/MoS_2_/FTO	84.56	0.1487	68.11	0.0913

#### 3.2.2 Effect of catalyst loadings

**Fig. 9:**
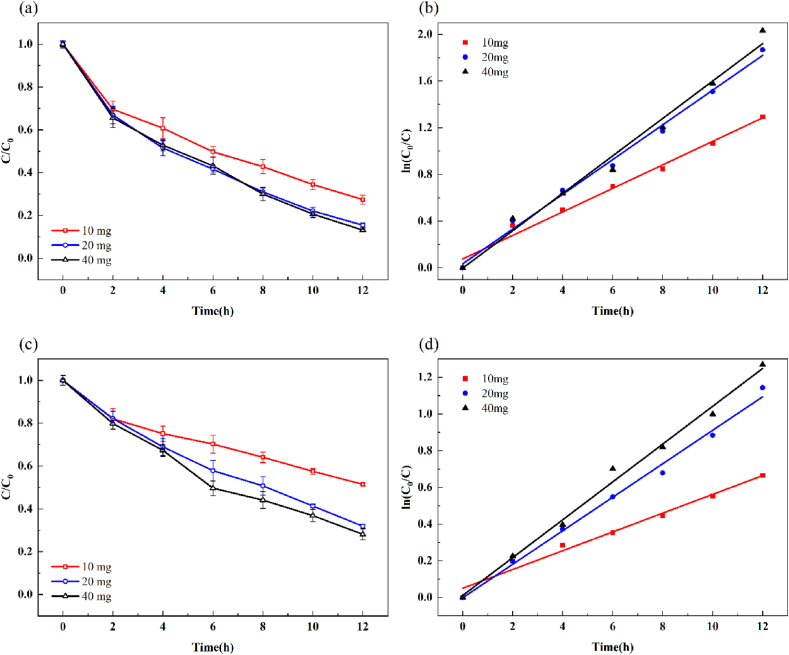
Effect of different catalyst loadings. (a) Degradation curve, (b) first order plots for the photodegradation of MB, (c) degradation curve, and (d) first order plots for the photoreduction of Cr(VI).

Fig. 9 shows the effect of cathodes with different WO_3_/MoS_2_ loadings on the removal of MB and Cr(VI). The removal efficiencies of MB and Cr(VI) with different catalyst loadings and their corresponding reaction rate constants are presented in Table 2. When the catalyst loading was increased from 10 to 20 mg, MB and Cr(VI) removal increased by 11.99 % and 19.54 %, respectively. However, when the catalyst loading was further increased to 40 mg, the MB and Cr(VI) removal rates increased by only 2.31 % and 3.77 %, respectively, owing to the limited surface area of FTO, which can only accommodate a certain number of active catalytic sites. Therefore, 20 mg was selected as the optimum catalyst loading, and subsequent experiments were performed at this level of addition.

**Table 2:** Removal and reaction rate constants of mixed pollutants of MB and Cr(VI) with different catalyst loadings.

Catalyst loading (mg)	MB		Cr(VI)	
	*η* (%)	k (h^−1^)	*η* (%)	k (h^−1^)
10	72.57	0.1007	48.57	0.0512
20	84.56	0.1487	68.11	0.0913
40	86.87	0.1602	71.87	0.1031

#### 3.2.3 Effect of pH

**Fig. 10:**
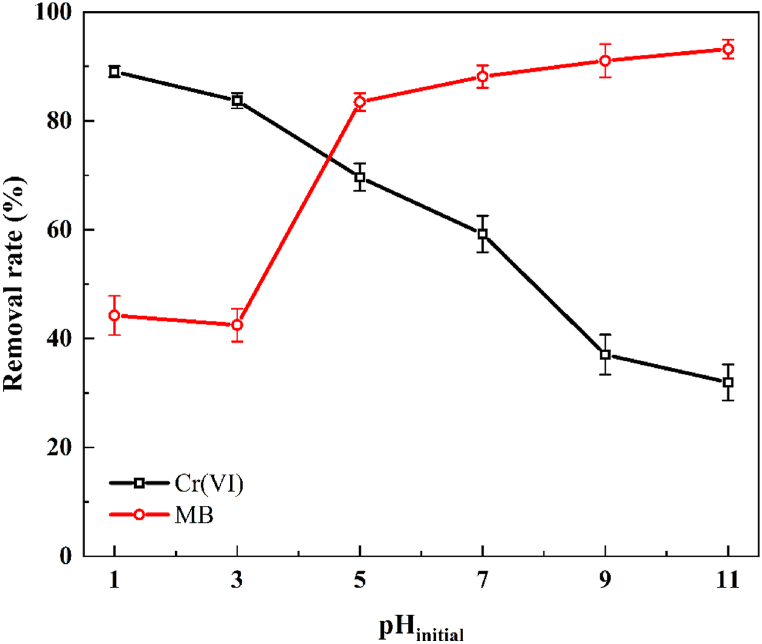
The effect of initial pH on the removal efficiency of MB and Cr(VI).

The pH at the active sites of the catalyst surface is a key determinant of reactive photocatalysis [42]. The pH of a mixed solution of 10 ppm MB and Cr(VI) was adjusted to 1, 3, 5, 7, 9, or 11 by adding NaOH or HCl. As shown in Fig. 10, when the pH was above 3, the removal rate of MB increased with increasing pH, whereas Cr(VI) showed the opposite trend, as higher pH values increased the number of negative charges on the WO_3_/MoS_2_/FTO surface due to protonation. Because MB is a cationic dye, it is attracted to negatively charged WO_3_/MoS_2_/FTO, which improves its removal rate [8,43]. Hexavalent chromium exists mainly as HCrO_4_^−^ under acidic conditions and as Cr_2_O_7_^2−^ in alkaline solutions. These negatively charged Cr(VI) species are electrostatically repelled by the negatively charged WO_3_/MoS_2_/FTO, thus reducing the Cr(VI) reduction rate. The removal of MB by WO_3_/MoS_2_/FTO exhibited the opposite trend when the pH was 1–3. This may be due to the fact that under strong acidic conditions, MB and HCrO_4_^−^ react to form flocculated chelates, reducing the number of adsorption sites for MB on the electrode surface [44]. Since the objective was to remove MB and Cr(VI) simultaneously, an initial pH of approximately 5 was selected for subsequent experiments.

#### 3.2.4 The relationship between the two pollutants

**Fig. 11:**
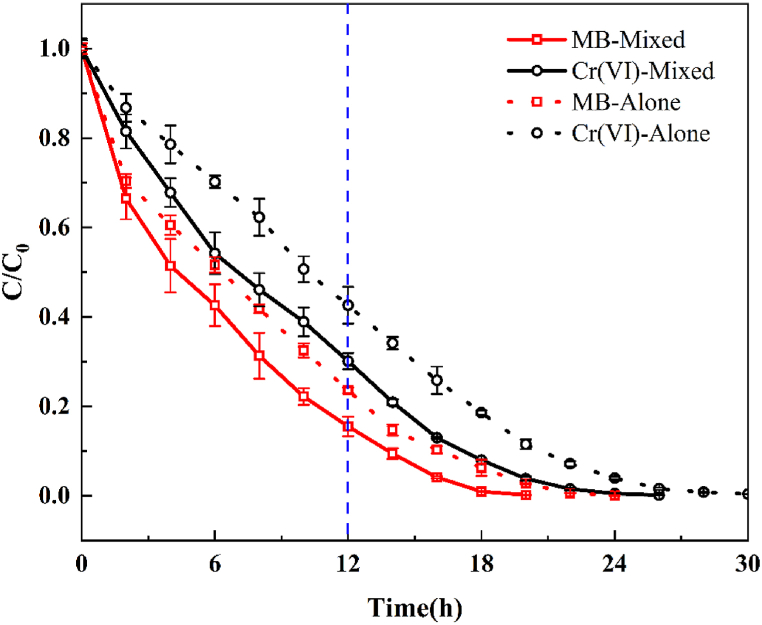
Removal efficiency of MB and Cr(VI) alone or mixed with WO_3_/MoS_2_/FTO.

To demonstrate the synergistic effect of mixing MB and Cr(VI) in the cathode chamber for their respective removal, MB, Cr(VI), and their mixtures were used as target pollutants. In the PMFC system, the photocathode was WO_3_/MoS_2_/FTO, the light source was 200 W LED light, the concentrations of the pollutants MB and Cr(VI) were both 10 ppm, and the initial pH of the solution was 5. Fig. 11 shows that after 12 h of reaction, the removal of MB and Cr(VI) from their respective solutions was 76.40 % and 57.39 %, respectively. In comparison, in the mixed solution, the MB and Cr(VI) removal rates increased by 8.16 % and 10.72 %, respectively. In terms of the time required for the complete removal of pollutants, the MB and Cr(VI) mixed pollutants were removed 4 h earlier than the corresponding single pollutants. This is because of a synergistic effect between MB and Cr(VI), where the mixed pollutants can simultaneously consume the electrons and holes photogenerated by WO_3_/MoS_2_/FTO under light irradiation, which is conducive to a reduction in the charge-carrier complexation rate, thus improving the removal efficiency of MB and Cr(VI) [45,46].

### 3.3 Photocatalytic mechanism

**Fig. 12:**
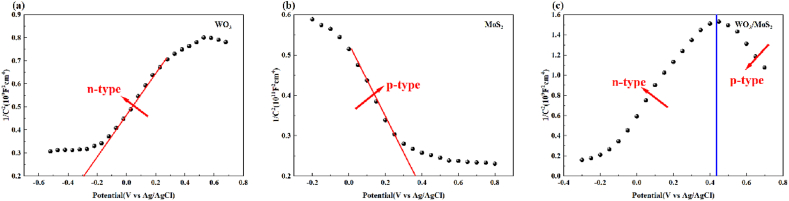
Mott–Schottky plots of (a) WO_3_ (n-type), (b) MoS_2_ (p-type), and (c) WO_3_/MoS_2_ composite (p-n heterojunction).

The conducting type and flat-band potential of the materials were determined using Mott–Schottky plots. As shown in Fig. 12 (a), the positive slope of WO_3_ indicates that it is an n-type semiconductor. Fig. 12 (b) shows a negative slope, indicating that MoS_2_ is a p-type semiconductor. When WO_3_ is combined with MoS_2_, an inverted "V-shaped" Mott–Schottky diagram is observed, which indicates that p-n heterojunctions with different electrical properties are formed between them [47,48], as shown in Fig. 12 (c). The intercept on the x-axis corresponds to the flat-band potential of the semiconductor material. The flat-band potentials of WO_3_ and MoS_2_ have estimated values E_fb_ = −0.29 V and 0.36 V with respect to Ag/AgCl, respectively [49,50].

**Fig. 13:**
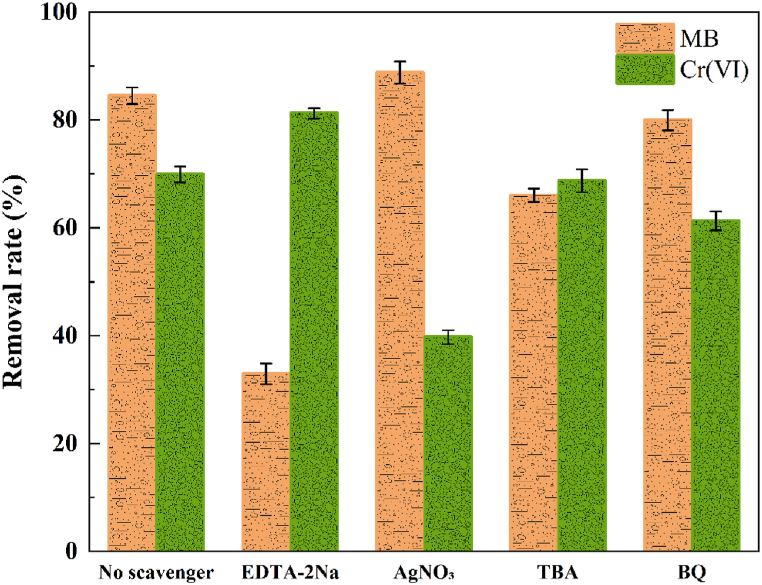
Removal rates of MB and Cr(VI) after adding various free-radical quenchers.

Free-radical trapping experiments were performed to investigate the roles of different active substances in pollutant removal. EDTA-2Na, AgNO_3_, tert-butanol, and *p*-benzoquinone were added to trap holes (h^+^), electrons (e^−^), hydroxyl radicals (•OH), and superoxide radicals (•O_2_^−^) formed during photocatalysis, respectively [51]. Fig. 13 shows the removal rates of MB and Cr(VI) with the addition of different radical trapping agents. By comparison to the absence of any radical traps, it was shown that the degradation of MB mainly depends on holes, followed by hydroxyl radicals, whereas superoxide radicals have little effect on degradation. When AgNO_3_ (e^−^ trapping agent) was added, the reduction rate of Cr(VI) was significantly reduced, whereas the addition of EDTA-2Na (h^+^ trapping agent) significantly enhanced the reduction rate. This indicates that the reduction of Cr(VI) is mainly caused by electrons because the consumption of holes reduces the loading rate of both electrons and holes and facilitates the directional transfer of electrons from the anode chamber to the cathode chamber, thereby increasing the reduction rate of Cr(VI).

**Fig. 14:**
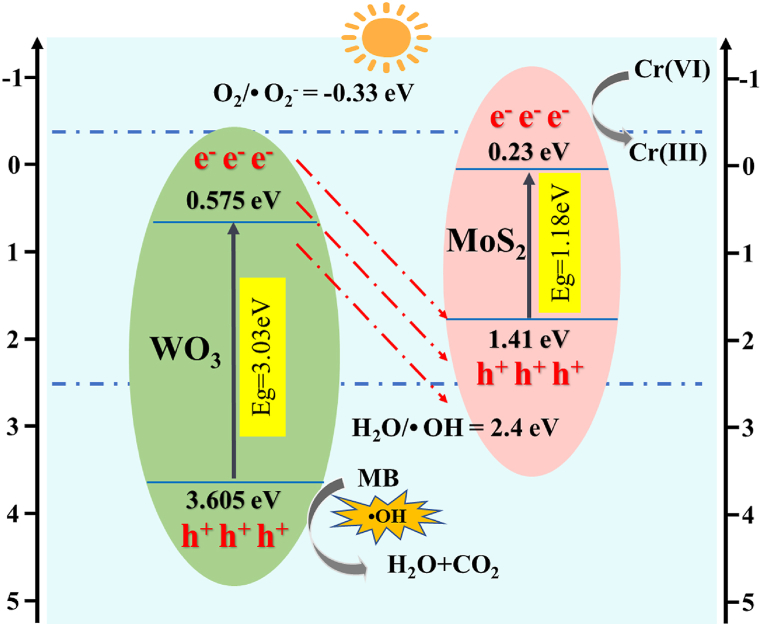
Plausible mechanism in the multifunctional PMFC system.

The working mechanism of the WO_3_/MoS_2_/FTO photocathode was analysed using UV–vis spectroscopy. The two main charge-transfer pathways involved in heterojunction photocatalysts are the conventional type-II heterojunction and direct Z-scheme heterojunction. As shown in Fig. 14, WO_3_ and MoS_2_ are excited simultaneously by light to produce photogenerated e^−^/h^+^. If WO_3_ and MoS_2_ form a type-II heterojunction, e^−^ on the conduction band of MoS_2_ will migrate to the conduction band of WO_3_, and h^+^ on the valence band of WO_3_ will transfer to the valence band of MoS_2_. Because the valence band potential of MoS_2_ is 1.41 eV, which is lower than the standard reduction potential of •OH/H_2_O (2.40 eV vs. NHE), no hydroxyl radicals can be generated from water [52]. The conduction band potential of WO_3_ is more positive than the standard reduction potential of O_2_/•O_2_^−^ (−0.33 eV vs. NHE), and therefore electrons here cannot convert oxygen to superoxide radicals. This assumption contradicts the results of the radical trapping experiments. Therefore, WO_3_ and MoS_2_ form a Z-scheme heterojunction, where e^−^ in the conduction band of WO_3_ combine with h^+^ in the valence band of MoS_2_, leaving a hole with a strong oxidising ability on WO_3_, which can further oxidise water to generate hydroxyl radicals, thus removing MB. This leaves reducing electrons in MoS_2_ to reduce Cr(VI). The combination of WO_3_ and MoS_2_ also pulls the already high conduction band to a higher position, which explains the appearance of a small number of superoxide radicals [53]. The specific reactions are summarised in Eqs. (10), (11), (12), (8), (9):

(8) $$\frac{{WO}_{3}}{M_{O}S_{2}}+hv\to {WO}_{3}\left(h_{VB}^{+}\right)+M_{O}S_{2}\;\left(e_{CB}^{-}\right)$$

(9) $${WO}_{3}\left(h_{VB}^{+}\right)+H_{2}O\to •OH+H^{+}$$

(10) $$M_{O}S_{2}\left(e_{CB}^{-}\right)+O_{2}\to •O_{2}^{-}$$

(11) $$M_{O}S_{2}\left(e_{CB}^{-}\right)+{Cr}_{2}O_{7}^{2-}+H^{+}\to {Cr}^{3+}+H_{2}O$$

(12) $${WO}_{3}\left(h_{VB}^{+}\right)/•OH/•O_{2}^{-}+Methylene\;blue\to \;CO_{2}+H_{2}O$$

In conclusion, the direct Z-scheme p-n heterojunction mechanism proposed for WO_3_/MoS_2_ composite photocatalysts improves the efficiency of photogenerated charge-carrier separation. It also provides a strong driving force for the photocatalytic degradation of organic pollutants and heavy metal reduction for environmental applications.

### 3.4 Stability study of photocathode WO_3_/MoS_2_/FTO in PMFC

**Fig. 15:**
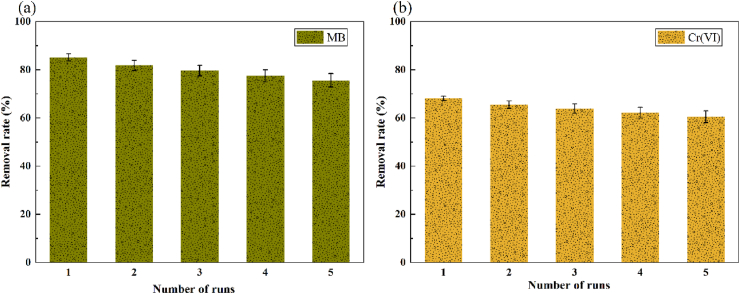
Simultaneous removal of (a) MB and (b) Cr(VI) in the PMFC under five light-operated cycles.

In large-scale practical applications, the lifetime of the photocathode must be considered; thus, the stability of the PMFC is an important indicator for evaluating cell performance. Fig. 15 shows the removal rates of MB and Cr(VI) mixed pollutants under continuous photocatalysis in the cathode chamber. After five cycles, the degradation rates of MB and Cr(VI) were maintained at 75.64 % and 60.53 %, respectively, indicating that WO_3_/MoS_2_/FTO has high stability and reproducibility, and great potential for application.

## 4 Conclusions

A PMFC system was established by coupling a bioanode with a WO_3_/MoS_2_/FTO photocathode to enhance pollutant removal and power generation. After 12 h of photocatalytic reaction, the removal of MB and Cr(VI) in the mixed solution was 84.56 % and 68.11 %, respectively, with a synergistic removal effect between the two pollutants. The maximum output power density of the cell was 33.14 mW/m^2^. Photoelectric test results showed that the PMFC had the lowest system internal resistance of 646.58 Ω when WO_3_/MoS_2_/FTO was the photocathode, and the photocurrent density was 1.0251 mA/m^2^, which was 1.79 times higher than that of WO_3_/FTO (0.5733 mA/m^2^). The formation of a p-n/Z-scheme heterojunction between WO_3_ and MoS_2_ reduces the recombination rate of photocarriers and enhances the removal of mixed MB and Cr(VI) pollutants.

## Data availability

The data generated and analysed in the current study are available from the corresponding author upon reasonable request. Owing to the nature of this research, the participants did not agree that their data would be publicly shared.

## CRediT authorship contribution statement

**Jiye Xin:** Writing – original draft, Project administration. **Shishi Kong:** Writing – review & editing, Data curation. **Xiaoliang Zhang:** Data curation. **Yujuan Yang:** Data curation. **Xuan Wang:** Writing – review & editing.

## Declaration of competing interest

We declare that we have no financial and personal relationships with other people or organizations that can inappropriately influence our work, there is no professional or other personal interest of any nature or kind in any product, service and/or company that could be construed as influencing the position presented in, or the review of, the manuscript entitled: “Simultaneous removal of MB and Cr(VI) in a dual-chamber photocatalytic microbial fuel with WO_3_/MoS_2_/FTO photocathode”.
